# Video-laryngoscopy introduction in a Sub-Saharan national teaching hospital: luxury or necessity?

**DOI:** 10.11604/pamj.2015.22.381.8175

**Published:** 2015-12-21

**Authors:** Traoré Ibrahim Alain, Barro Sié Drissa, Kaboré Flavien, Ilboudo Serge, Traoré Idriss

**Affiliations:** 1 Anesthesiology and Reanimation Department, Bobo-Dioulasso University, Burkina-Faso; 2 Anesthesiology and reanimation Department, Ouagadougou University, Burkina-Faso

**Keywords:** Video-laryngoscopy, difficult tracheal intubation, sub-saharan teaching hospital

## Abstract

Tracheal intubation using Macintosh blade is the technique of choice for the liberation of airways. It can turn out to be difficult, causing severe complications which can entail the prognosis for survival or the adjournment of the surgical operation. The video-laryngoscope allows a better display of the larynx and a good exposure of the glottis and then making tracheal intubation simpler compared with a conventional laryngoscope. It is little spread in sub-Saharan Africa and more particularly in Burkina Faso because of its high cost. We report our first experiences of use of the video-laryngoscope through two cases of difficult tracheal intubation which had required the adjournment of the interventions. It results that the video-laryngoscope makes tracheal intubation easier even in it's the first use because of the good glottal display which it gives and because its allows apprenticeship easy. Therefore, it is not a luxury to have it in our therapeutic arsenal.

## Introduction

Tracheal intubation using the Macintosh laryngoscope is frequently realized at the University teaching hospital Souro Sanou (CHUSS) of Bobo-Dioulasso for the liberation of the airway when mechanical ventilation is recommended. It is the technique of choice in this type of indication but it can turn out to be difficult even impossible to be realized. It is then at the origin of severe complications able to entail the prognosis for survival or the adjournment of the surgical operation. The video-laryngoscope, developed for more than a decade, allows a better display of the larynx and a good exposure of the glottis, so making tracheal intubation simpler compared with a conventional laryngoscope [1–3]. It enters the algorithm of difficult intubations according to international recommendations [4]. Nevertheless, this new technique of indirect laryngoscopy is little spread in sub-Saharan Africa and more particularly in Burkina Faso because of its high cost. We here report our first experience of use of the video-laryngoscope through two cases of difficult tracheal intubation which had required the adjournment of intervention for lack of alternatives.

## Patient and observation

**Figure 1:**
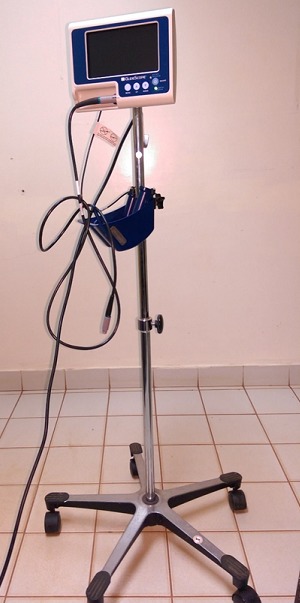
Vidéo-laryngoscope (Glidescope)

**Observation 1**: it was about a 50-year-old patient without a particular pathological history which had to benefit from the removal of a right maxillary tumor at the University teaching hospital Souro-Sanou of Bobo-Dioulasso. Three weeks previously, the patient had been programmed for the same intervention. Consultation in anesthesia revealed at the time criteria of difficult intubation with, in particular, a score of Mallampati of 4, a limitation of the oral opening of 2,5 cms and a partial toothless just sparing tooth number 31,41 and 42. Thyro-mental distance was superior to 6,5 cms. In the operating room, after monitoring (electrocardiograph, non invasive blood pressure, saturation of 02) and a preoxygenation in the hanging facial mask for 3 minutes, the induction of anesthesia was done with 120 mg of propofol, 30 mg of suxamethonium chloride and 9γ of sufentanyl. The tracheal intubation was unsuccessfully tried by a male nurse anaesthetist then a doctor in the second year of specialization in anesthesia resuscitation with a number 4 blade of the Macintosh laryngoscope. In the laryngoscopy the doctor could not visualize the epiglottis in spite of various external laryngeal operations undertaken. After oxygenation and reinjection of 30 mg of suxamethonium chloride, a second laryngoscopy was three times unsuccessfully undertaken by a senior doctor anaesthetist (5 years of experience). The adjournment of the surgical operation was then decided. Three weeks later, the hospital, having received a video-laryngoscope, the patient was again programmed. The same anesthetic protocol as well as a monitoring identical to the first intervention was established. The laryngoscopy was then realized by means of video-laryngoscope Glidescope ^R^ (Figure 1) by a doctor anaesthetist having never used this technique, under the supervision of another regular visitor familiar with the video-laryngoscope. Tracheal intubation was then realized in one minute without incident with a good display of all the epiglottis in the laryngoscopy (Cormack 1).

**Observation 2**: it was about an 18-year-old patient without a particular pathological history who had to benefit from a liberation of scar reins at the level of the neck and of the face after effects of thermal burn of the face. The patient weighed 50kg and was 1,60 m tall. He had already been programmed for the same intervention two months ago. Preanesthetic consultation at the time revealed criteria of difficult intubation, in particular a thyro-mental distance of 5 cms, an oral opening of 2,5cms and a score of Mallampati of 3. After monitoring (electrocardiograph, no invasive blood pressure, saturation of 02) and preoxygenation, the induction of anesthesia had been done by propofol 100 mg and suxamethonium chlorid 50 mg. The intubation had been tried on 3 occasions by a senior doctor anaesthetist using a Machintosh blade 4 with no visualization of the glottis. Facing desaturation of 90%, a laryngeal mask number 4 was then set up. The intervention was postponed in absence of other alternatives. Two months later, the patient was reprogrammed for the same surgical operation. A protocol of induction similar to the first intervention was realized. Tracheal intubation was again tried by another senior doctor anaesthetist with a Macintosh blade 4. In spite of 2 attempts and external laryngeal operations, the laryngoscopy objectified only a rank 4 of Cormack and Léhane. After oxygenation and reinjection of 50 mg of suxamethonium chlorid, the intubation was easily realized in two minutes by the same doctor anaesthetist by using Glidescope ^R^. In the laryngoscopy we noted a rank 1 of Cormack and Léhane.

## Discussion

**Figure 2:**
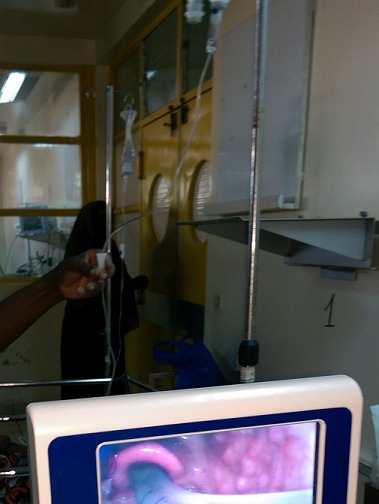
Image of the passage of the probe of intubation at the level of vocal cords during the use of the video-laryngoscope

The incapacity to maintain the freedom of airways after induction of general anesthesia is a major concern for all anaesthetists. To reassure air traffics, tracheal intubation using direct laryngoscopy remains the method of choice in most of the cases. However, in direct laryngoscopy intubation can be difficult in 1-4% of the cases and the impossible in 0, 05-0,35% of the patients of the general population [5]. In case of difficult or impossible intubation, the difficulty of preservation of the permeability of airways can cause severe complications such as hypoxic brain damages or death [6]. Several alternative techniques in case of difficult intubation via direct laryngoscopy by a Macintosh blade exist. Among these techniques, the standard gold is the use of supple fiberscope which is however expensive and of more difficult manipulation [7]. The video laryngoscope Glidescope ^R^ type, became since then a reliable alternative in case of difficult or impossible intubation. It allows a good display of the glottis when it is bad or invisible in conventional laryngoscopy [1, 2]. In our patients, during attempts of intubation, conventional laryngoscopy showed only a rank IV of Cormack and Léhane whereas Glidescope ^R^ had allowed an excellent display of the glottis with a cormack I (Figure 2). This difference of display of the glottis is understandable by the fact that the Glidescope because of its configuration does not require a compulsory alignment of the bucco pharynx axes for the laryngoscopy [8–12]. Indeed, the blade of Glidescope ^R^ contrary to that of the Macintosh laryngoscope possesses an additional angulation in 60° upward distal half. This enables to insert the blade along the median line of the tongue, to follow the superior air traffics without any movement of the tongue and without necessity of aligning the optical axes. Other studies also demonstrated that Glidescope ^R^ reduced the difficulty of tracheal intubation compared with the Macintosh laryngoscope when used by experimented anaesthetists as well as by beginners [10, 11]. In our first patients, Glidescope ^R^, used by a doctor in the course of specialization in anesthesia/resuscitation, thus little experimented regarding tracheal intubation had all the time allowed a complete display of the glottis and an easy intubation without incident. Furthermore, these studies revealed that Glidescope ^R^ reduced the time required to make a tracheal intubation as well as the risk of dental trauma. It was also considered easier to use than the Macintosh laryngoscope during normal and difficult intubations. The video-laryngoscope is thus a necessity in our context where most of the tracheal intubations are realized by male nurse anaesthetists having a less good control of direct laryngoscopy compared with doctor's anaesthetists. The video-laryngoscope will also allow to teach in a more effective way laryngoscopy to male nurse anaesthetists but also to doctors and students. For the next decade, video-laryngoscopes are probably going to be used in first intention for tracheal intubation. It is consequently essential to begin to get acquainted with them. Their relatively prohibitive prices and their maintenance limit their popularization in all the situations of intubation in Burkina Faso for the moment. However, due to their performance in the management of difficult airways, their availability in various university teaching hospitals for difficult intubation, their use becomes rational and deeply wished.

## Conclusion

These clinical cases represent the first publication on the use of the video-laryngoscope in Burkina Faso. It results from them that the video-laryngoscope makes tracheal intubation easier even for the first use because of the good glottal display which it gives and of apprenticeship easier as well. It is thus not luxury to have it in our therapeutic arsenal. Its relatively high cost can constitute a barrier for its popularization in all our operating rooms. It would be interesting at first to set it up in the university teaching hospitals of the country. Doctor anaesthetists in training, student nurse anaesthetists as well as medical students can also learn this technique which, during the next decade, is probably going to become the technique of first intention for routine tracheal intubation.
